# Investigation of Grain Refinement Mechanism of Nickel Single Crystal during High Pressure Torsion by Crystal Plasticity Modeling

**DOI:** 10.3390/ma12030351

**Published:** 2019-01-23

**Authors:** Peitang Wei, Hao Zhou, Huaiju Liu, Caichao Zhu, Wei Wang, Guanyu Deng

**Affiliations:** 1 State Key Laboratory of Mechanical Transmissions, Chongqing University, Chongqing 400044, China; 20142143@cqu.edu.cn (H.Z.); huaijuliu@cqu.edu.cn (H.L.); cczhu@cqu.edu.cn (C.Z.); weiwang_cqu@cqu.edu.cn (W.W.); 2 School of Mechanical, Materials, Mechatronic and Biomedical Engineering, University of Wollongong, Wollongong NSW 2522, Australia; gd577@uowmail.edu.au

**Keywords:** high pressure torsion, crystal plasticity, grain refinement, microstructure, lattice rotation

## Abstract

The excellent properties of ultra-fine grained (UFG) materials are relevant to substantial grain refinement and the corresponding induced small grains delineated by high-angle grain boundaries. The present study aims to understand the grain refinement mechanism by examining the nickel single crystal processed by high pressure torsion (HPT), a severe plastic deformation method to produce UFG materials based upon crystal plasticity finite element (CPFEM) simulations. The predicted grain maps by the developed CPFEM model are capable of capturing the prominent characteristics associated with grain refinement in HPT. The evolution of the orientation of structural elements and the rotations of crystal lattices during the HPT process of the detected differently oriented grains are extensively examined. It has been found that there are mainly two intrinsic origins of lattice rotation which cause the initial single crystal to subdivide. The correlation between the crystallographic orientation changes and lattice rotations with the grain fragmentation are analyzed and discussed in detail based on the theory of crystal plasticity.

## 1. Introduction

Conventional heavy deformation processes, such as rolling, drawing and extrusion, can result in significant grain refinement, but the structures formed usually have low angle grain boundaries. Ultra-fine grained (UFG) materials processed by severe plastic deformation (SPD) processes, especially high pressure torsion (HPT), however, are characterized by smaller grains that are mainly surrounded by high-angle grain boundaries (HAGBs) [1,2,3]. It has been well known that the excellent properties of UFG materials such as strength, wear resistance, fatigue, and fracture properties are relevant to the substantially small grains and HAGBs [4,5,6]. Therefore, an accurate description of the grain refinement process and formation of high-angle boundaries in HPT can deep the understanding of the underlying deformation mechanisms, and will facilitate the development of a new generation of UFG materials.

The commonly accepted type of grain refinement mechanism due to large strain induced by HPT is based on the notion that a dislocation cell structure, which forms in the early stages of plastic deformation, gradually transforms to the final fine grain structure [4,7,8,9]. This is believed to occur through a continual decrease in the average grain size accompanied by the accumulation of misorientation between neighboring dislocation cells. Recently, the two-dislocation density model, where the dislocation cell walls and cell interiors were considered as separate ‘phases’, has been employed to simulate the HPT process. For instance, Estrin et al. [10,11] applied such a model to explain the occurrence of a uniform microstructure as a result of an inherently non-uniform deformation during the HPT process. Lee et al. [12] used this model to investigate the cell/grain size evolution with the accumulation of dislocation density. The simulations have proved that the two-dislocation density model was a good tool for predicting the evolution of the microstructure in HPT.

An alternative explanation for grain refinement is the source–sink mechanism of dislocations [13,14,15]. During deformation, the dislocations glide through the grain and contribute to local deformation. When the dislocation reaches the boundary region it can be trapped or annihilated by other dislocations, producing misorientation between the adjacent grain fragments. Horita and his co-workers investigated the microstructural evolution of various HPT-processed high purity metals and alloys [2,16,17]. They proposed that the grain-refining mechanism in HPT follows the microstructural evolution as accumulation of dislocations, formation of subgrain boundaries, enhancement of misorientation angle, absorption of dislocations at high-angle boundaries, and establishment of a steady state due to a balance between the dislocation generation and absorption at high-angle boundaries.

On the other hand, lots of experimental observations favored the intergranular gliding of dislocations, and the lattice rotation promoted the fragmentation of the microstructure during HPT [3,18,19,20,21]. Different sets of slip systems would be activated in different regions of a grain to maintain the deformation compatibility, thus leading to strong heterogeneities in the local lattice rotation within the single grain. The formation of grain boundaries is accompanied by the rotation of individual structural elements. Crystal plasticity modelling, which is based on the assumption of crystallographic slip and stretching and rotating of the crystal lattice when the crystalline material undergoes deformation, provides a potential way to explore the underlying mechanism of grain refinement, as reported for KOBO extrusion [22], cyclic expansion–extrusion [23], equal channel angular pressing process [24], etc. However, for the HPT process, very few studies associated with crystal plasticity have been reported in the literature. Hafok and Pippan [25] and Wei et al. [26] applied the fully constrained Taylor model and the crystal plasticity model to predict the texture evolution of nickel single crystal during HPT, respectively, but did not research the grain-refinement process. Accepting that HPT shearing is achieved by intergranular glide, Kratochvíl and his co-authors [27,28] interpreted the fragmentation process in HPT within the framework of crystal plasticity. They found that the effective rotation of the double slip and the local variations in the crystal lattice orientation were mainly responsible for the microstructure fragmentation. The proposed crystal plasticity model was a simplified version based on the assumption of uniform deformation of plane-strain carried by double slip, and provided limited understanding of the grain refinement behavior for HPT.

In this study, a three-dimensional crystal plasticity finite element (CPFE) model taking into account the evolution of the orientation of structural elements and rotations of crystal lattices of crystallites was developed to simulate the grain fragmentation of nickel single crystal during the HPT process. The single crystal was chosen because it eliminates the effect of grain boundary and grain-grain interaction. Grain maps detected at different stages of HPT deformation were presented. The development processes of crystallographic orientation changes and lattice rotation of the detected grains were examined in detail. The main purpose of this work was to study how an original single crystal fragments into many different oriented grains after HPT deformation and to explore the underlying mechanism.

## 2. CPFE Simulation Model

### 2.1. Crystal Plasticity Theory

The detailed descriptions of the kinematical scheme and constitutive relations used in this study can be found in References [29,30].

The crystalline slip is assumed to follow the power law, and the resolved shear strain rate ${\dot{\gamma }}^{\left(\alpha \right)}$ on a slip system is uniquely determined by the resolved shear stress $\tau^{\left(\alpha \right)}$ as follows:

(1) $${\dot{\gamma }}^{\left(\alpha \right)}={\dot{\gamma }}_{0}^{\left(\alpha \right)}sgn\left(\tau^{\left(\alpha \right)}\right){\left|\frac{\tau^{\left(\alpha \right)}}{\tau_{c}^{\left(\alpha \right)}}\right|}^{n}\;\mathrm{for}\;\left|\tau^{\left(\alpha \right)}\right|\gg \tau_{c}^{\left(\alpha \right)}\;sgn\left(x\right)=\{\begin{array}{c} -1,\;x<0\\ 1,\;x\geq 0 \end{array}$$

where ${\dot{\gamma }}_{0}^{\left(\alpha \right)}$ and n are the material parameters, $\tau_{c}^{\left(\alpha \right)}$ is the critical resolved shear stress of the slip system α.

When more than one slip system is active, hardening in each slip system is a function of the slip on all the active slip systems; the rate of increase of the flow stress $\tau_{c}^{\left(\alpha \right)}$ is therefore specified as:

(2) $${\dot{\tau }}_{c}^{\left(\alpha \right)}=\overset{N}{\underset{\beta =1}{\sum }}h_{\alpha \beta }{\dot{\gamma }}^{\left(\beta \right)}$$

where the matrix $h_{\alpha \beta }$ contains the hardening moduli for each slip system. $h_{\alpha \alpha }$ is known as self-hardening while $h_{\alpha \beta }$ is known as latent hardening.

The Bassani and Wu model [31], which was based upon the characterization of hardening moduli at any stage during deformation, was utilized in this work. It has been proved by a great number of researches associated with crystal plasticity that Bassani and Wu’s model could reflect the hardening of face-centered cubic crystals (fcc) more exactly [32,33,34]. The expressions for self and latent hardening take the following form:

(3) $$\begin{array}{c} h_{\alpha \alpha }=\left[\left(h_{0}-h_{s}\right){\sec h}^{2}\left(\frac{\left(h_{0}-h_{s}\right)\gamma^{\left(\alpha \right)}}{\tau_{1}-\tau_{0}}\right)+h_{s}\right]\left[1+\sum_{\begin{array}{c} \beta =1\\ \beta \neq \alpha \end{array}}^{N}f_{\alpha \beta }\tan h\left(\frac{\gamma^{\left(\beta \right)}}{\gamma_{0}}\right)\right]\;\mathrm{for}\;\alpha =\beta \\ h_{\alpha \beta }=qh_{\alpha \alpha }\;\mathrm{for}\;\alpha \neq \beta \end{array}$$

where *q* is a latent hardening parameter. $h_{0}$, $h_{s}$, $\tau_{0}$, and $\tau_{s}$ are hardening moduli and shear stresses. γ stands for the shear strain. $f_{\alpha \beta }$ denotes the interaction parameter between the two slip systems α and β. The factors $f_{\alpha \beta }$ depend on the geometric relation between two slip systems. There are five constants for $f_{\alpha \beta }$, namely $\alpha_{1}$ (no junction), $\alpha_{2}$ (Hirth lock), $\alpha_{3}$ (coplanar junction), $\alpha_{4}$ (glissile junction), and $\alpha_{5}$ (sessile junction).

### 2.2. Crystal Plasticity Finite Element Model

**Figure 1:**
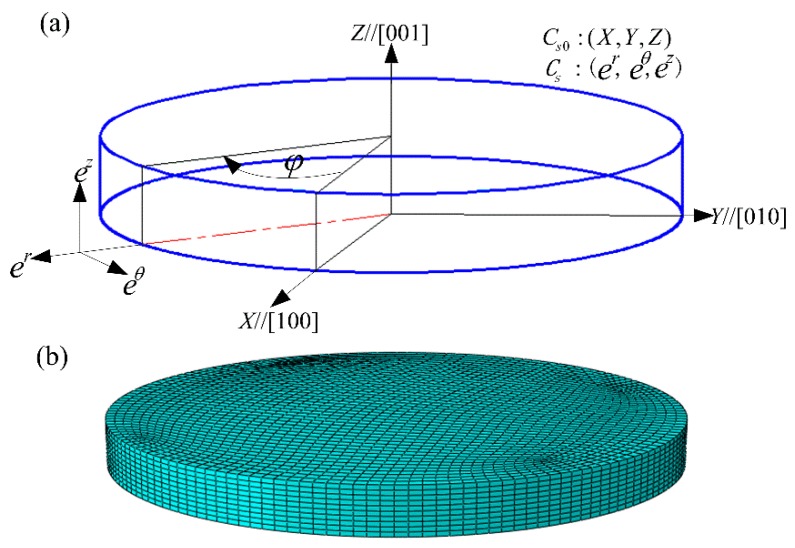
CPFE model of HPT [35]. (**a**) Configuration of the sample and the initial orientation; (**b**) meshes of the sample.

The CPFE model of HPT was constructed using the commercial software Abaqus/Standard ver. 6.9-1, as schematically illustrated in Figure 1. The initial dimensions of the deformable disk-shaped sample were 10 mm in diameter by 0.8 mm in thickness, while the upper and lower HPT anvils were set to rigid bodies. The sample consisted of nickel single crystal which was initially oriented with (001) crystallographic plane normal parallel to the *Z* axis and [100] crystallographic direction lying with the *X* axis of the global coordinate system. Since HPT deformation is exerted on the small metallic disc, a local polar coordinate system $C_{S0}$ was established with the associated orthonormal base vector ($e^{r},\;e^{\theta },\;e^{z}$), where *R*, θ and *Z* represent the radial, tangential and axial directions at a given location, respectively. The angular values φ stand for the rotation degrees with respect to the *X* axis around the axial direction on the top surface, as illustrated in Figure 1a. The linear brick element with reduced integration C3D8R was the type of element chosen for the sample, and 23,600 elements in total were generated, as shown in Figure 1b. During the simulation, the lower anvil was fixed, while the rotation boundary condition along the *Z* axis was applied to the upper anvil with the other freedoms of the upper anvil set to be constrained.

The parameters in the constitutive equations of the simulated nickel material are listed in Table 1. These parameters have been validated in our previous work of CPFEM modelling of texture evolution during the HPT process [26]. The crystal plasticity constitutive model was implemented implicitly in Abaqus/Standard adopting the user material subroutine (UMAT), where the material properties can be defined and the data can be exchanged with Abaqus. UMAT calculated the Jacobian matrix of the current material configuration and updated the predefined state variables such as the stress state, crystallographic orientations, etc. CPFE simulations were based on 12 {111}〈110〉 slip systems in the case of fcc crystals.

### 2.3. Grain Structure Reconstruction Algorithm

**Figure 2:**
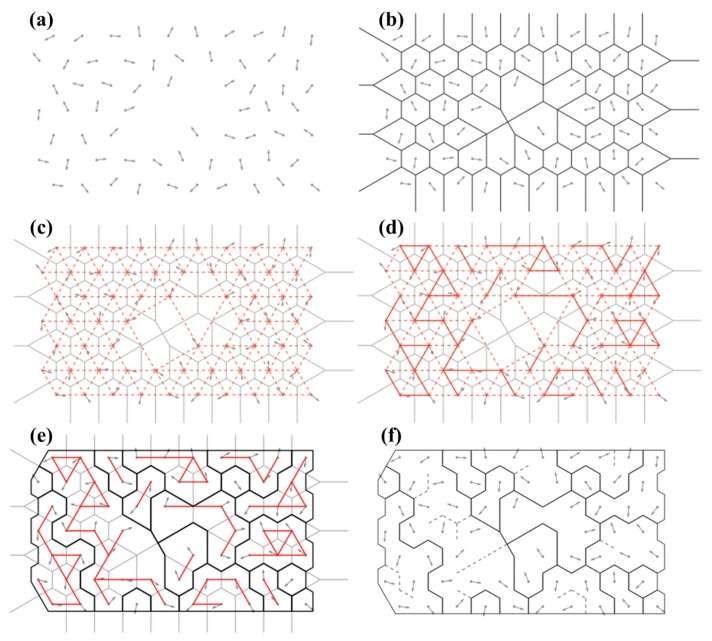
Schematic diagram of the grain structure reconstruction algorithm [36]. (**a**) The orientation data, (**b**) the Voronoi decomposition of the inspection locations, (**c**) the neighborhood relationships of Voronoi cells, (**d**) adjacent Voronoi cells that are not separated by a grain boundary (**e**) adjacent Voronoi cells that are separated by a grain boundary and (**f**) resulting partition displaying grain boundaries.

In this study, the grain structure reconstruction algorithm proposed by Bachmann et al. [36] was adopted, as schematically illustrated in Figure 2, the principle procedures of which are described as follows:

(1) The resultant data including the inspection locations and predicted orientations collected from the interested two-dimensional domain were imported for preparation, as shown in Figure 2a, where the predicted locations were marked by black dots and their corresponding orientations were displayed as directions.

(2) Voronoi decompositions of the inspection locations were conducted, which resulted in a series of Voronoi cells carrying their own orientations, as depicted in Figure 2b. Information regarding the vertices and edges of the cells were stored in the corresponding incidence matrices.

(3) These incidence matrices were used to find all the adjacency pairs, i.e., the positions of potential grain boundaries. These neighborhood relationships are illustrated in Figure 2c, where the neighboring Voronoi cells are linked by hatched red lines.

(4) Subsequently, the common edge between two adjacent Voronoi cells was checked to judge whether it is an actual grain boundary. The common edge was identified as being within the same grain if the corresponding misorientation angle, which refers to the minimum angle $\theta_{min}$ among all the crystallographic equivalent rotations that bring the lattices of adjacent grains into coincidence, is smaller than a given threshold angle $\theta_{c}$. The adjacent cells that are not separated by a grain boundary are linked together by bold red lines, as shown in Figure 2d. Otherwise, if $\theta_{min}>\theta_{c}$, the common edge is determined as a grain boundary. The grain boundaries between two neighboring cells are denoted by bold black lines, as shown in Figure 2e.

(5) A grain was defined as a region in which the misorientation of at least one neighboring inspection location is smaller than the threshold angle $\theta_{c}$. By following this grain characterization rule, all the generated grains could be detected, as shown in Figure 2f.

**Figure 3:**
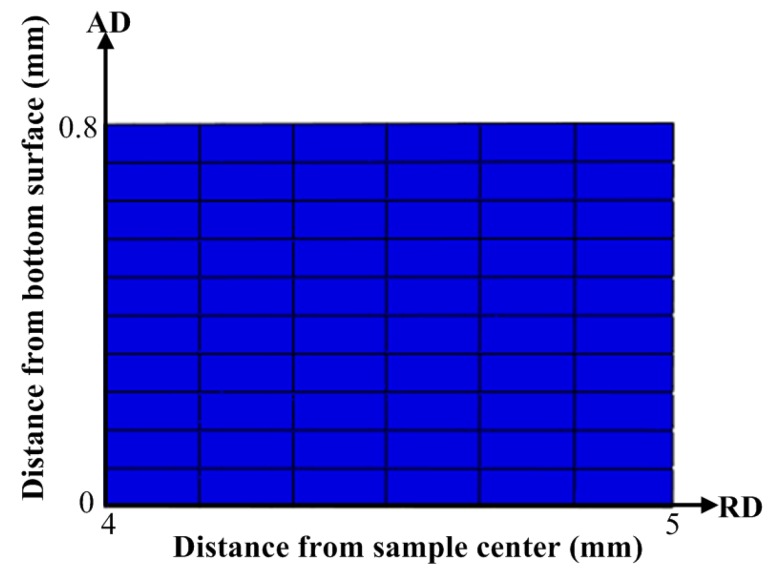
Schematic description of the region selected for grain detection.

A rectangular region located at *φ* = 75° along the circumferential direction was chosen as a representative to record the reconstructed grain maps during the HPT deformation, as shown in Figure 3. The shear direction of material points within the selected region is identical, hence it is convenient for the obtained results to be expressed in the local polar coordinate system. The horizontal direction was the radial direction (RD) while the vertical direction referred to the axial direction (AD). The length of the rectangular region spread from 4 to 5 mm from the center of the sample and the width covered the whole thickness of the sample. In the selected region, there were RD = 6 and AD = 10 meshes in the radial and axial direction, respectively. Potentially, these meshes permit fragmentation into a maximum of RD X AD grains in the studied cross-section. The meshes used here are expected to be too coarse to represent the grain-scale deformation. However, we do expect the meshes to be able to capture the prominent features associated with grain fragmentation during the HPT process.

## 3. Results and Discussion

### 3.1. Detection and Description of Grain Refinement

**Figure 4:**
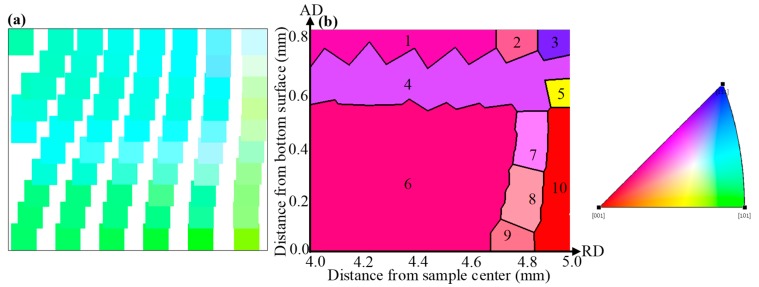
The predicted grain map recorded on the selected *R*-*Z* plane after (**a**) *N* = 1/16 turn HPT deformation, (**b**) *N* = 1/8 turn HPT deformation.

Figure 4a,b plots the predicted grain subdivision states of the initial nickel single crystal subjected to HPT deformations of *N* = 1/16 turn and 1/8 turn, respectively. The color of each individual grain was determined by its calculated mean orientation, as illustrated by the unit triangle. HAGBs with misorientation larger than 15° were recorded and depicted by black lines.

As can be seen in Figure 4a, the HAGB is not formed yet within the studied cross section after *N* = 1/16 turn HPT deformation. However, the continuous changes in the color, namely the crystallographic orientation, from the top to the bottom surface is clearly visible. This result obtained by the developed CPFEM model is in good agreement with the experimental observation of Pippan et al. [18]. They experimentally examined the orientation map of a copper single crystal deformed by HPT to a similar strain level to the present work, and found that the measured grain color changed gradually along the axial direction.

After *N* = 1/16 turn HPT deformation, Figure 4b indicates that grain subdivision has already taken place in the original single crystal. On the left side of the map, three lamellar-shaped substructures delineated by HAGBs could be observed. The major axes of the detected substructures are almost parallel to the radial direction. The simulated microstructure morphology is consistent with the experimental observations made by Orlov et al. [8]. They researched the microstructural characteristics in pure aluminum during HPT and revealed that the HPT deformation-induced grain were lamellar and clearly elongated along the radial direction on the *R*-*Z* plane at low strain. Also, Barnett et al. [20,37] showed by using a Taylor type model that high-angle boundaries produced by torsion are likely to have a disorientation axis parallel to the sample radial direction. Additionally, a few differently oriented grains appear at the sample periphery ranging between 4.7 and 5 mm in the sample radius, wherein more plastic strain is introduced. The morphology and size of these grains differ significantly; namely, some grains retain the lamellar structure whereas other substructures are further deformed to the smaller portions.

### 3.2. Mechanisms of Grain Refinement

In order to understand how one single crystal initially fragments into many differently oriented substructures, the predicted grain map after *N* = 1/8 turn HPT deformation was examined in detail. For convenience, the detected grains in Figure 4b were designated as Grain 1 to Grain 10, respectively. To help interpret the grain refinement process, investigations into the crystallographic orientation changes and lattice rotations along the radial and axial directions, respectively, were carried out. All resultant pole figures showing the crystal orientation changes were recorded on *θ*-*Z* planes. In each pole figure, the initial orientation and orientation after deformation were denoted by red asterisks and thick dots of different colors, respectively. The locations of the main components of the ideal torsion texture for FCC metals were also visualized using different symbols. The lattice rotation angles of detected grains relative to the initial orientations were calculated and partitioned into three components which represent the rotation angles around the *R*, *θ* and *Z* axes, respectively. The positive value means a counter-clockwise lattice rotation, while the negative value means a clockwise rotation.

**Figure 5:**
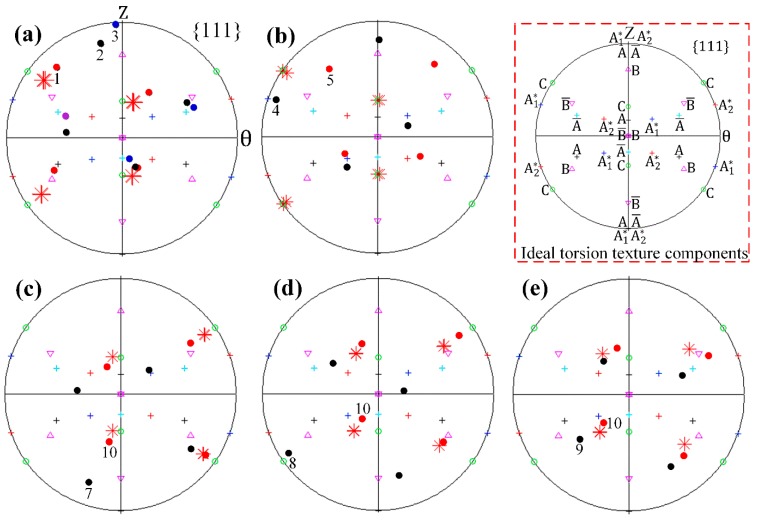
The predicted {111} pole figures of the detected grains along the radial direction. (**a**) Grains 1, 2 and 3, (**b**) Grains 4 and 5, (**c**) Grains 7 and 10, (**d**) Grains 8 and 10, (**e**) Grains 9 and 10.

**Figure 6:**
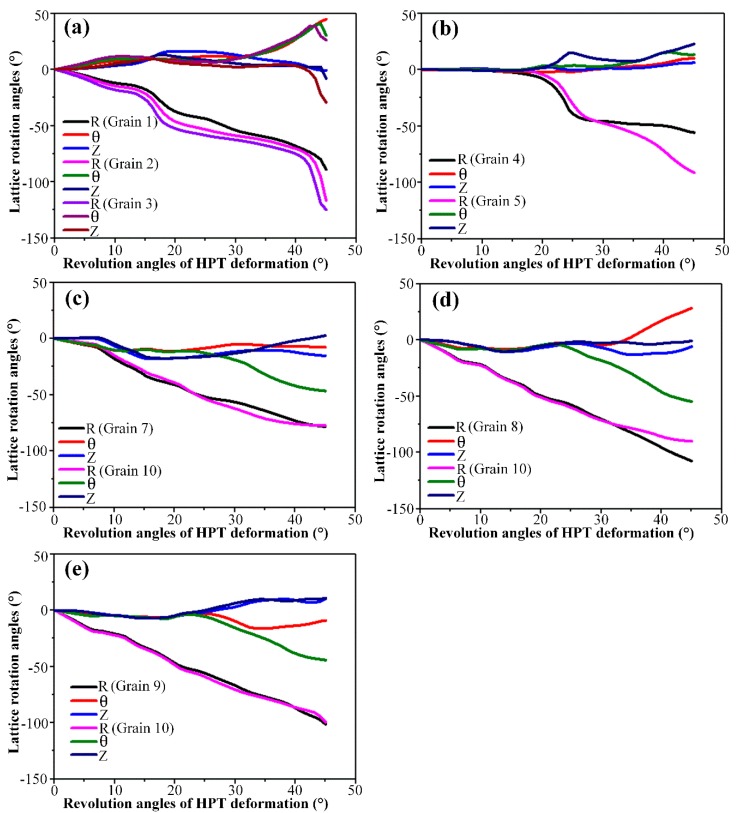
The predicted lattice rotation angles of the detected grains along the radial direction. (**a**) Grains 1, 2 and 3, (**b**) Grains 4 and 5, (**c**) Grains 7 and 10, (**d**) Grains 8 and 10 and (**e**) Grains 9 and 10.

The generated neighboring grains along the radial direction at various sample thicknesses were comparatively analyzed in terms of {111} pole figures and lattice rotations, as shown in Figure 5 and Figure 6, respectively. It is seen from Figure 5 and Figure 6 that the grains selected for analysis have almost the same initial orientations close to the *C* component of the ideal torsion texture, and all lattices rotate predominately along the radial axis to accommodate the increasingly introduced plastic strain. After 45° (*N* = 1/8 turn) HPT deformation, the dominant *R*-axis lattice rotation angles in Figure 6a exhibit obvious divergences due to the strain gradient existing along the radial direction, which is assumed to be responsible for the formation of Grains 1, 2 and 3 (Figure 5a). Such a grain fragmentation phenomena is supported by the experimental observations and numerical simulations as reported in References [20,27,28]. Barnett and Montheillet [20] carried out a series of torsion tests on aluminum to investigate the generation mechanism of new high-angle boundaries during torsion. Their results indicated that high-angle boundaries of deformation in torsion can be ascribed to sub-grain rotation, and these high-angle boundaries were characterized by a disorientation axis close to parallel with the sample radial direction. Kratochvíl et al. [27,28] showed by a crystal plasticity-based model of microstructure evolution during HPT that the most distinguished feature relating to the HPT microstructure in the thickness direction was the radial gradient of shear strain, and the effective rotation of crystal slip systems along the radial direction led to the microstructure fragmentation of materials deformed by HPT. Grain 3 has an orientation close to the ideal $\bar{A}$ component which aligns the $\left(\bar{1}\bar{1}1\right)$ crystallographic plane with the shear plane and is, therefore, colored in blue in the predicted grain map of Figure 4, while the orientations of Grains 1 and 2 are rotating towards the same preferred orientation of $\bar{A}$ at distinctly varying degrees. Similarly, it is revealed that there is ≈ 40° variation in the *R*-axis lattice rotation in Figure 6b, which give rise to the development of Grains 4 and 5 in this partial region. The orientation of generated Grain 4 is identified to be close to the $A_{1}^{*}$ component while the poles of Grain 5 occupy positions greatly away from the $A_{1}^{*}$ in the corresponding pole figure of Figure 5b.

For the formation of HAGBs of the rest of the thickness positions, the functions of lattice rotation around the *R*, *θ* and *Z* axes have changed significantly. The dominant *R*-axis lattice rotations shown in Figure 6c–e illustrate basically no divergence between adjacent radial positions. Even so, it is observed that the resultant crystallographic orientations of the adjacent radial positions occupy obviously different locations in the corresponding pole figures of Figure 5c–e, indicating the occurrence of grain fragmentation. Further examinations of Figure 6c–e reveal that a noticeable amount of lattice rotation along the θ axis also occur, and are accompanied by the predominant *R*-axis lattice rotations, specifically, which are ≈ −5° of Grain 7, ≈ 25° of Grain 8, ≈ −10° of Grain 9 and ≈ −45° of Grain 10. Therefore, it can be stated that the formation of HAGBs that bounded Grains 7 and 10, Grains 8 and 10 as well as Grains 9 and 10 are mainly attributed to the distinct variation of lattice rotation lying on the *θ* axis.

**Figure 7:**
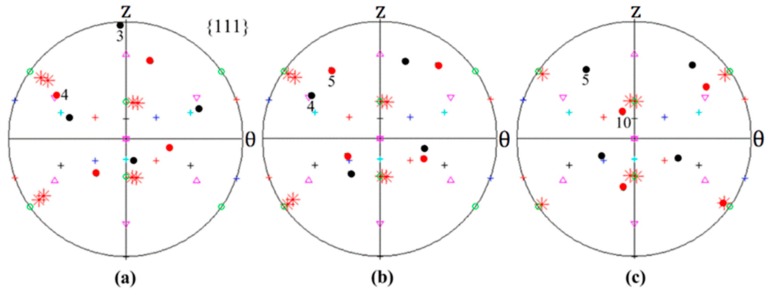
The predicted {111} pole figures of the detected grains along the axial direction. (**a**) Grains 3 and 4, (**b**) Grains 4 and 5 and (**c**) Grains 5 and 10.

**Figure 8:**
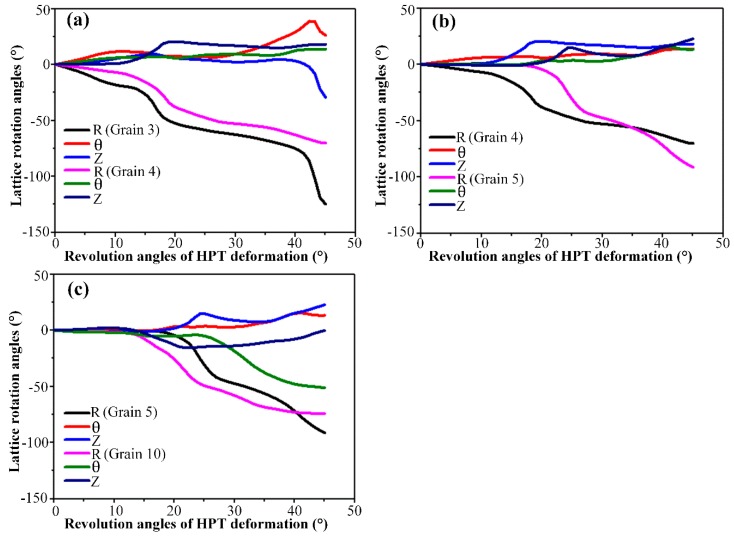
The predicted lattice rotation angles of the detected grains along the axial direction. (**a**) Grains 3 and 4, (**b**) Grains 4 and 5 and (**c**) Grains 5 and 10.

In this section, the adjacent grains along the axial direction positioned close to the sample periphery, namely Grains 3 and 4, Grains 4 and 5 as well as Grains 5 and 10 were selected for comparative analysis. The same method used in the radial direction analysis was adopted. The resultant {111} pole figures and lattice rotations are presented in Figure 7 and Figure 8, respectively. In each pole figure of Figure 7, one can see that the initial orientations of the detected grains are slightly different due to the intrinsic properties of the disk-shaped single crystal and the inherent characteristics of material flow on the shear plane [38].

The lattice rotation results around all three axes recorded in Figure 8a illustrate similar tendencies as those of Figure 5a. After 45° HPT deformation, the divergence in *R*-axis lattice rotation becomes relatively visible with an amount of ≈ 50°, which contribute to the formation of the distinctly misoriented Grains 3 and 4 along the axial direction. It can be seen from Figure 7a that Grain 3 has an orientation close to the $\bar{A}$ component of the ideal torsion texture while the orientation of Grain 4 has just rotated away from the ideal $A_{1}^{*}$ component. Also, such a divergence in the *R*-axis lattice rotation causes Grains 4 and 5 to form, as shown in Figure 7b. Moreover, close to the bottom surface, it is obvious from Figure 8c that the opposite sense lattice rotation along the θ axis is mainly responsible for the formation of the two different sharp orientation branches, Grains 5 and 10, in this region. As can be observed in Figure 7c, the orientation of Grain 5 has rotated further away from the ideal $A_{1}^{*}$ component, while the orientation of Grain 10 occupies positions close to the ideal *C* component and is accordingly colored in red in Figure 4.

**Figure 9:**
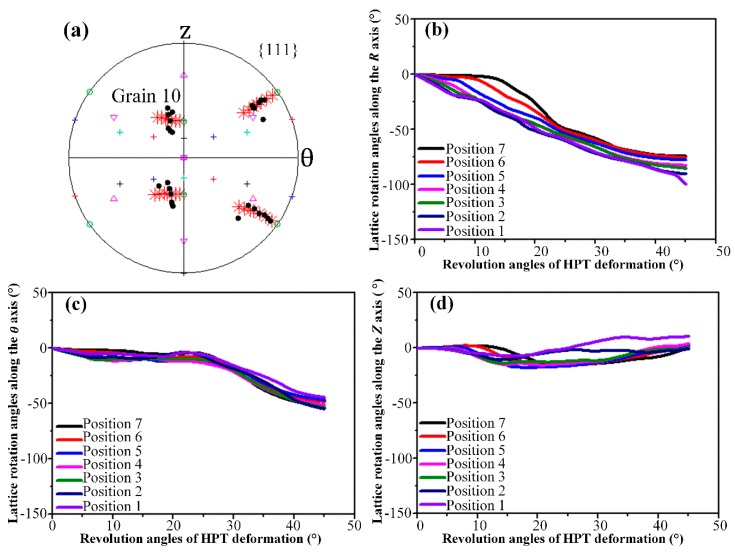
The predicted results for various thickness positions of Grain 10. (**a**) {111} pole figure, (**b**) lattice rotation angles along the *R* axis, (**c**) lattice rotation angles along the θ axis and (**d**) lattice rotation angles along the *Z* axis.

At the lower right corner of the predicted grain map in Figure 4, only one large grain of Grain 10 with a major axis parallel to the axial direction could be detected. As can be seen in Figure 9a, under an undeformed condition, there is a clear initial orientation gradient in this region. However, such an initial orientation gradient does not lead to grain fragmentation after 45° HPT deformation as described in Figure 5 and Figure 7. To explain this phenomenon, the lattice rotations around all three axes of various thickness positions within Grain 10 were calculated and shown in Figure 9b–d. Position 1 corresponds to the axial location close to the bottom surface and it extends to position 7 along the axial direction as surface thickness increases. As can be seen, from position 7 to position 1, the lattice rotation angles around the *R* and *θ* axes increase gradually at any straining moment till 45° HPT deformation. That is to say, the bulk region undergoes similar bulk reorientation after deformation. As a consequence, the pole figure of Grain 10 reveals the developed orientations spread about a single average orientation of an ideal *C* component rather than splitting into multiple orientation branches.

### 3.3. Discussion

It is well established form the previous studies that lattice rotation plays a key role in the development of HAGBs and fragmentation of grains in HPT (e.g., References [3,18,38,39,40,41]). The rigid body rotation field generated by the HPT process produces local lattice rotations within the single crystal, which produce ever greater numbers of HAGBs, eventually generating small, rotated grains. The characteristics of lattice rotation during the large strain HPT/torsion deformation have also been investigated by many experimental observations and numerical simulations [20,26,42,43]. From the quoted literature, some common features of lattice rotation phenomena during HPT deformation can be identified: One is the predominance of single sense rotations around the sample radial direction and another is the location of regions of crystallographic divergence adjacent to the main deformation texture components. Nevertheless, it remains unclear how the lattice rotation causes the initially uniformly oriented crystals subdividing into portions with a range of different orientations. To answer this question, the grain subdivision mechanism in HPT was explored with the aid of the crystallographic orientation changes and the lattice rotation obtained by the developed CPFEM model. It is found that there are two main lattice rotation modes under which grain boundaries with high misorientations could be developed.

On one hand, the main reason for grain subdivision lies in the dominant *R*-axis lattice rotation. The divergence in rotation rate along the radial direction promotes the formation of widely different oriented grains, which is supported by the development processes of Grains 1, 2 and 3, Grains 4 and 5 along the radial direction as well as Grains 3 and 4 and Grains 4 and 5 along the axial direction. Such a process is also confirmed by the work of Barnett and Montheillet by investigating the generation of new HAGBs in aluminum during torsion deformation based upon texture measurements and a Taylor-type model [20,37]. They reported that the new high-angle boundaries were associated with a subgrain rotating away from the parent grain in the single sense around the radial direction. The reason for the occurrence of divergence in *R*-axis rotation rate is that the lattice rotates non-uniformly with respect to the uniformly introduced shear deformation in HPT. Tóth et al. [42,44] demonstrated by using the crystallographic rotation field that the lattice rotation rate is significantly large at positions rotating way from the main ideal torsion texture components, whereas it decreases asymptotically while approaching the ideal ones.

On the other hand, it is of interest to notice that the small lattice rotation along the θ axis could serve as the intrinsic origin that facilitates the generation of grains separated by boundaries of high misorientations. In such circumstances, though the *R*-axis lattice rotation still dominates the HPT straining process, its influence on grain subdivision could be basically neglected due to the relatively small divergence between adjoining segments. The *θ*-axis lattice rotation-induced grain fragmentation can be widely observed in this study, such as the developed HAGBs that surrounded Grains 7 and 10 as well as Grains 5 and 10. It is particularly obvious in the pole figure of Figure 8c, in which the grain subdivision is evidently achieved by the moderate amount of rotations but in an opposite sense about the θ axis. This result is in good accord with the prediction of the reorientation field divergence theory model reported by Raabe et al. [45]. The results (Figure 3a and Figure 6c) of Reference [45] revealed that the grain with initial Goss orientation had split into two different sharp orientation branches during straining, which are related to each other by a rotation about the transverse direction, corresponding to the θ direction of the present work.

## 4. Conclusions

In the present work, a CPFE model was established to study the grain refinement behaviour of nickel single crystal during the HPT process. The grain maps obtained by the developed CPFE model were capable of describing the prominent characteristics associated with grain refinement during the HPT process. The development process over a set of differently oriented grains bounded by high-angle boundaries on the predicted grain map were extensively examined by the combination of local lattice rotations and orientation changes to explore the underlying grain refinement mechanism. It has been found that there were mainly two intrinsic origins of lattice rotation which caused the initial nickel single crystal to subdivide: One is the difference in the rotation rate along the radial direction, which is the direction of predominant lattice rotation in HPT. The other one is the divergence of rotation angles along the θ axis; even though they are much smaller than the *R*-axis rotations, they can also contribute to the formation of grains with significantly large misorientations.

## Figures and Tables

**Table 1:** Parameters in the hardening law.

*C*_11_ (MPa)	*C*_12_ (MPa)	*C*_44_ (MPa)	*n*	${\dot{\gamma }}_{0}$ (s^−1^)	*h*_0_ (MPa)	*h_s_*
246500	147300	124700	50	0.001	61.8	0.01
$\tau_{1}$ (MPa)	$\tau_{0}$ (MPa)	$\alpha_{1}$	$\alpha_{2}$	$\alpha_{3}$	$\alpha_{4}$	$\alpha_{5}$
26.7	17.5	0.4	0.4	0.4	0.75	1
